# Diagnostic, Therapeutic, and Prognostic Value of the m^6^A Writer Complex in Hepatocellular Carcinoma

**DOI:** 10.3389/fcell.2022.822011

**Published:** 2022-02-09

**Authors:** Zongting Gu, Yongxing Du, Xueping Zhao, Chengfeng Wang

**Affiliations:** ^1^ Department of Abdominal Surgery, National Cancer Center/Cancer Hospital, Chinese Academy of Medical Sciences and Peking Union Medical College, Beijing, China; ^2^ School of Life Science and Biopharmaceutical, Shenyang Pharmaceutical University, Shenyang, China

**Keywords:** M6A, RNA methylation, writer complex, methyltransferase, immune infiltration, hepatocellular carcinoma

## Abstract

Hepatocellular carcinoma (HCC) has poor prognosis and is usually diagnosed only at an advanced stage. Identification of novel biomarkers is critical to early diagnosis and better prognosis for HCC patients. N6-methyladenosine (m^6^A) RNA methylation regulators play important roles in the development of many tumors. However, the m^6^A writer complex, a key executor of m^6^A methylation modification, has not been independently investigated, and its specific bioinformatics analysis has not yet been performed in HCC. In this study, we used multiple public databases to evaluate the diagnostic, therapeutic, and prognostic value of the m^6^A writers in HCC. The results showed that expression levels of METTL3, VIRMA and CBLL1 were significantly increased, while expression levels of METTL14 and ZC3H13 were significantly decreased in HCC, which was closely related to clinicopathological factors, such as tumor stage and prognosis. Bioinformatics further explored the possible underlying mechanisms by which the m^6^A writer complex are involved in activation of tumor-promoting pathways and/or inhibition of tumor-suppressing pathways, including apoptosis, cell cycle, DNA damage response and EMT. Furthermore, we showed that the m^6^A writer complex is correlated with immune cell infiltration and immunoregulator expression in HCC. In conclusion, the m^6^A writer complex may represent a promising biomarker and target that can guide targeted therapy or immunotherapy for HCC patients.

## Introduction

Globally, liver cancer is the fourth leading cause of cancer-related deaths, of which hepatocellular carcinoma (HCC) accounts for more than 80%, resulting in a heavy burden of disease (Global Burden of Disease Cancer et al., 2019). Over the past few decades, although considerable progress has been made in the epidemiology, risk factors, and molecular mechanisms of HCC, the incidence and cancer-specific mortality in many countries continue to increase, which is related to the fact that most HCC patients are diagnosed at an advanced stage and lack effective treatment options (Villanueva, 2019). Consequently, it is urgent to clarify the specific mechanism of HCC to develop novel biomarkers, improve the rate of early diagnosis, and identify new targets for molecular targeted therapy.

Epigenetic modifications are involved in the onset and progression of human diseases,especially cancer (Gu et al., 2015; Bauer et al., 2016). Various genetic and epigenetic alterations in hepatocytes result in the conversion of proto-oncogenes into oncogenes and the loss of tumor suppressor genes, ultimately promoting carcinogenesis and progression of HCC (Shibata, 2021). The molecular mechanisms associated with genetics, including chromosomal translocations, single nucleotide polymorphisms and loss or deletion of targeted genes, and epigenetic modifications, including gene-specific DNA methylation modifications, aberrant histone modifications, have been extensively explored in HCC (Shibata, 2021). However, as a novel epigenetic modification, the role of RNA methylation in cancer, especially in HCC, has not been fully defined, which has given rise to a new field of research called “epitranscriptomics” (Saletore et al., 2012).

**FIGURE 1:**
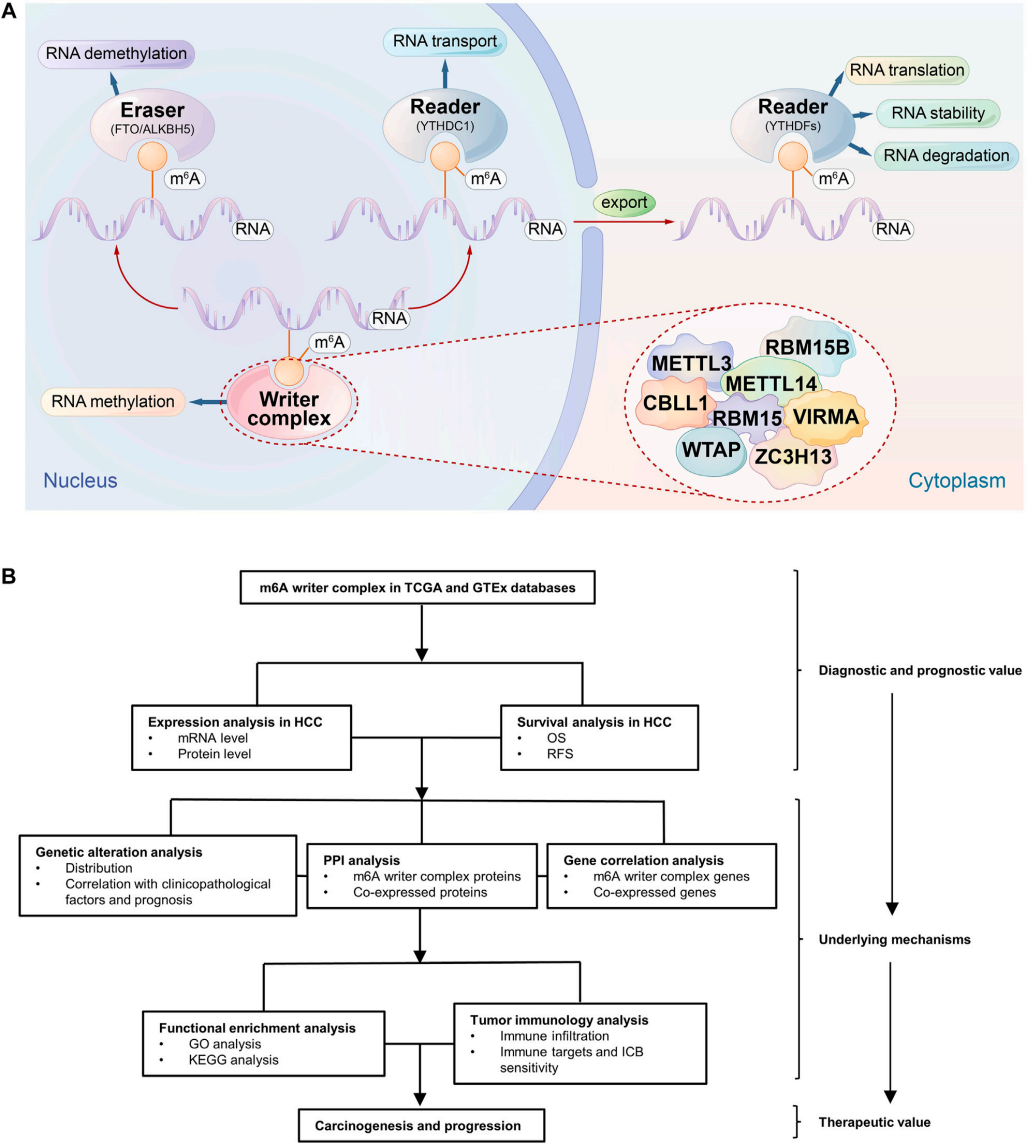
Schematic diagram of the study. **(A)** The m^6^A mRNA life cycle. m^6^A methylation is a dynamic and reversible process involving methyltransferases (writers), removal by demethylases (erasers), and binding to specific reader proteins that affect the stability, translation and degradation of mRNA. The m^6^A writer complex consists of METTL3, METTL14, WTAP, RBM15, METTL15B, VIRMA, ZC3H13, and CBLL1. **(B)** The flowchart of the study.

N6-methyladenosine (m^6^A) refers to the methylation modification of the sixth nitrogen (N) atom of adenine (A), which accounts for more than 60% of RNA modifications, especially the modification of eukaryotic mRNA, and affects the RNA metabolism, such as splicing, transport, translation, and degradation (Yu et al., 2018). Accumulating evidence suggests that dysregulated m^6^A modification is involved in the carcinogenesis and progression of multiple cancers; for example, dysregulated m^6^A modification in the transcripts of some oncogenes, such as Snail, or tumor suppressor genes, such as PHLPP2, is associated with tumor proliferation and metastasis (Liu et al., 2018a; Lin et al., 2019), and they have the potential for targeted therapies (Su et al., 2018). Notably, the current role of m^6^A modification in cancer seems to be conflicting. Some genes promote tumor development after methylation, while others can promote tumor development after the removal of methylation (Ma et al., 2017; Chen et al., 2018). In addition, multiple studies have revealed a correlation between infiltrating immune cells in the tumor microenvironment (TME) and m6A modification (Li et al., 2020a; Zhang et al., 2020a), which may affect the response to immune checkpoint blocking (ICB) therapies (Xu et al., 2021). Therefore, m^6^A modification may play a regulatory role in the tumorigenesis, progression and immune regulation of HCC. The m^6^A modification in mRNA is reversible and dynamically regulated by methyltransferase (writer), demethylase (eraser), and binding protein (reader). However, the tumor-promoting or tumor-suppressive roles of these three regulators are not consistent (Melstrom and Chen, 2020). In fact, writers are a type of protein complex with m6A methyltransferase catalytic activity, i.e., a writer complex. Its components include METTL3, METTL14, WTAP, RBM15/15B, VIRMA, ZC3H13 and CBLL1, as well as other possible components (Gu et al., 2020), which catalyze m^6^A formation in the mRNA of oncogenes or tumor suppressors and trigger a series of molecular biological effects, which in turn regulates the expression of cancer-related genes (Zaccara et al., 2019) (Figure 1A). Accordingly, as the initiator of methylation modification, the function and regulation of writer complex components will be key to understanding the nature and function of regulated m^6^A sites. However, the relationship between the writer complex and HCC is still unclear, and relevant studies are also scarce.

Based on this background, our study focused on the analysis of m^6^A writer complex-related genes (Figure 1B). Using TCGA and GTEx databases, we compared writer complex expression and prognosis differences between HCC samples and matched normal liver tissues. The possible mechanisms of these genes involved in the tumorigenesis and development of HCC were further explored by the analysis of gene alterations, protein interactions, functional enrichment and immune infiltration. Importantly, we validated the results in different databases to increase the credibility of the results. The findings of this study will help to identify potential diagnostic markers and novel targets for treatment, guide early clinical diagnosis and individualized treatment, and improve the prognosis of patients with HCC.

## Materials and Methods

### Ethics Statement

This study was approved by the ethics committee of the National *Cancer* Center/*Cancer* Hospital, Chinese Academy of Medical Sciences (Beijing, China) and was performed in accordance with the principles of the Declaration of Helsinki. All datasets for this study are freely available from the published literature and do not involve any human or animal experiments.

### Expression Analysis

Based on TCGA and GTEx databases, Oncomine (Rhodes et al., 2004) (https://www.oncomine.org/) and GEPIA (Tang et al., 2017) (http://gepia.cancer-pku.cn/) online tools were used to visualize differences in gene expression in the m^6^A writer complex between HCC and adjacent normal tissues, and the two results were mutually verified. We used UALCAN (Chandrashekar et al., 2017) (http://ualcan.path.uab.edu/) and GEPIA online tools to compare the relationship between m^6^A writer complex expression and HCC stage and pathological grade. We used R software (version 3.6.3) to evaluate the relationship between the m^6^A writer complex and other clinicopathological features. We utilized the HPA(Asplund et al., 2012) (https://www.proteinatlas.org/) database to analyze protein expression levels of the m^6^A writer complex in HCC.

### Survival Analysis

We used the Kaplan–Meier Plotter (Győrffy et al., 2013) (www.kmplot.com) online tool to analyze the correlation between the expression of the m^6^A writer complex genes and overall survival (OS) and relapse-free survival (RFS) in HCC. The split cutoff of low and high expression was set in the auto select best cutoff model, and biased arrays were excluded. The log-rank test was used to compute the *p*-value, and *p* < 0.05 was regarded as significant.

### Genetic Alteration Analysis

We used the cBioPortal (Cerami et al., 2012) (https://www.cbioportal.org/) database to analyze genetic alterations in the m^6^A writer complex and further determined the correlation between mutation and several important clinicopathological factors and survival (Gao et al., 2013).

### Correlation and Interaction Analysis

We applied the TIMER (Li et al., 2017) (http://timer.cistrome.org/) database to analyze the correlation in gene expression between the m^6^A writer-complex components in HCC and then plotted the heatmap based on the Pearson correlation coefficient. The volcano map of differentially expressed genes related to the m^6^A writer complex in HCC and the heatmap of the top 50 genes positively/negatively correlated with the m^6^A writer complex were drawn using the LinkedOmics (Vasaikar et al., 2018) (http://www.linkedomics.org/) database. A heatmap of the correlation between the m^6^A writer-complex components based on protein expression data was obtained by combined score analysis in the STRING (Mering et al., 2003) (https://string-db.org/) database. In addition, we used the igraph package (version 1.2.6) and ggraph package (version 2.0.5) of R software (version 3.6.3) to construct a network of the m^6^A writer complex and the 10 most frequently altered coexpressed genes. The protein–protein interaction (PPI) network of the m^6^A writer complex was constructed using the STRING database and visualized using Cytoscape software (Shannon et al., 2003; Doncheva et al., 2019) (v3.9.0), and then the cytoHubba plug-in (Chin et al., 2014) (http://apps.cytoscape.org/apps/cytohubba) was used to screen the top 10 hub genes based on degree value rank.

### Functional Enrichment Analysis

We applied the Metascape (Zhou et al., 2019) (https://metascape.org) database to explore functional enrichment of the hub genes, while pathway enrichment was performed using the GSCALite (Liu et al., 2018b) (http://bioinfo.life.hust.edu.cn/web/GSCALite/) online tool. Gene Ontology (GO) and Kyoto Encyclopedia of Genes and Genomes (KEGG) analyses of the m^6^A writer complex and coexpressed genes were performed using the ClusterProfiler package (Yu et al., 2012) (version 3.14.3) in R for functional annotation and pathway enrichment, respectively. GO analysis included biological process (BP), cellular component (CC) and molecular functions (MF).

### Tumor Immunology Analysis

We applied R’s GSVA package (Hänzelmann et al., 2013) (version 1.34.0) based on the TCGA database, combined with the TISIDB (Ru et al., 2019) (http://cis.hku.hk/TISIDB/index.php) database to analyze the relationship between the m^6^A writer complex and immune cell infiltration in HCC. In addition, TISIDB was used to analyze the relationship between the m^6^A writer complex and expression of immunomodulators in HCC. We analyzed the correlation between writer complex expression and drug sensitivity in immune or targeted therapies by applying the GSCALite online tool based on the GDSC database (Yang et al., 2013).

## Results

### Transcriptional Levels of the m^6^A Writer Complex in Hepatocellular Carcinoma

**FIGURE 2:**
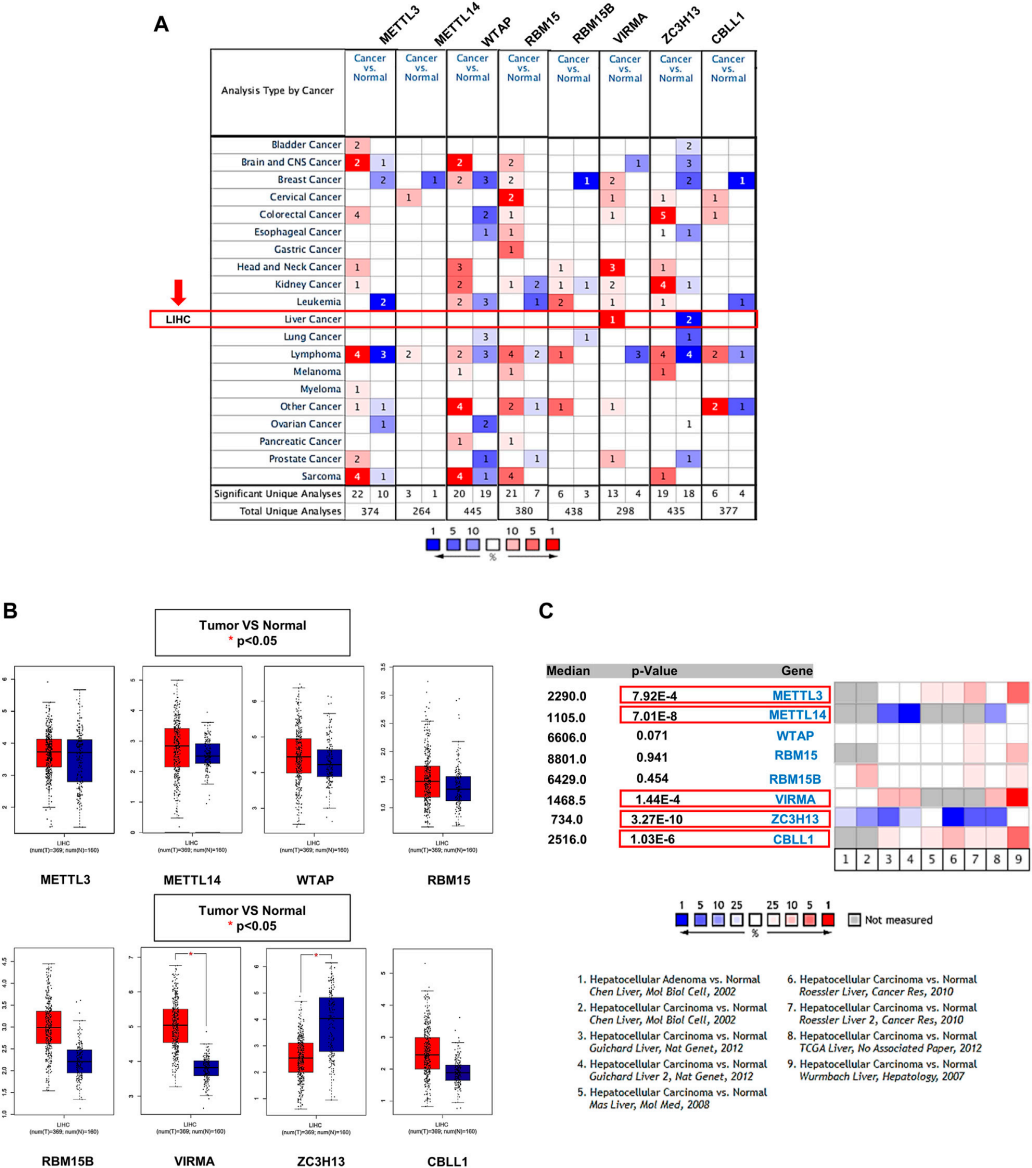
Transcriptional levels of the m^6^A writer complex in HCC. **(A)** mRNA expression levels of the m^6^A writer complex (Oncomine). The box indicated by the red arrow shows liver hepatocellular carcinoma (LIHC). The colored squares represent the median rank of these genes (vs. normal tissue). Red represents high expression and blue represents low expression. Differences in transcriptional expression were compared using Student’s t-test. The cutoff *p*-value and fold change were as follows: *p*-value: 0.01, fold change: 1.5, gene rank: 10%, data type: mRNA. **(B)** Box plot of m^6^A writer complex expression in HCC (GEPIA). Red represents the expression in HCC tissue, and blue represents the expression in normal tissue. *p < 0.05. **(C)** Meta-analysis of the mRNA expression levels of the m^6^A writer complex using the nine Oncomine datasets. The colored squares represent the median rank of these genes (vs. normal tissue) across the nine datasets. Red represents high expression and blue represents low expression. The significance level for the median rank analysis was set at *p* < 0.05.

The Oncomine database showed that gene expression of the m^6^A writer complex in cancer tissues is different from that in normal tissues, but not all components exhibited similar changes (Figure 2A). Among them, VIRMA and ZC3H13 exhibited increased and decreased transcription levels in HCC, respectively, compared to normal tissues (Figure 2A). Results from the GEPIA database also confirmed the differential expression of VIRMA and ZC3H13 in HCC (Figures 2B,C). Of concern, a subset of datasets in the Oncomine also exhibited significantly higher expression levels of METTL3 and CBLL1 and lower expression levels of METTL14 in HCC compared to normal tissues, although there was no significant difference in TCGA database (Figure 2D).

### Tissue Levels of the m^6^A Writer Complex in Hepatocellular Carcinoma

**FIGURE 3:**
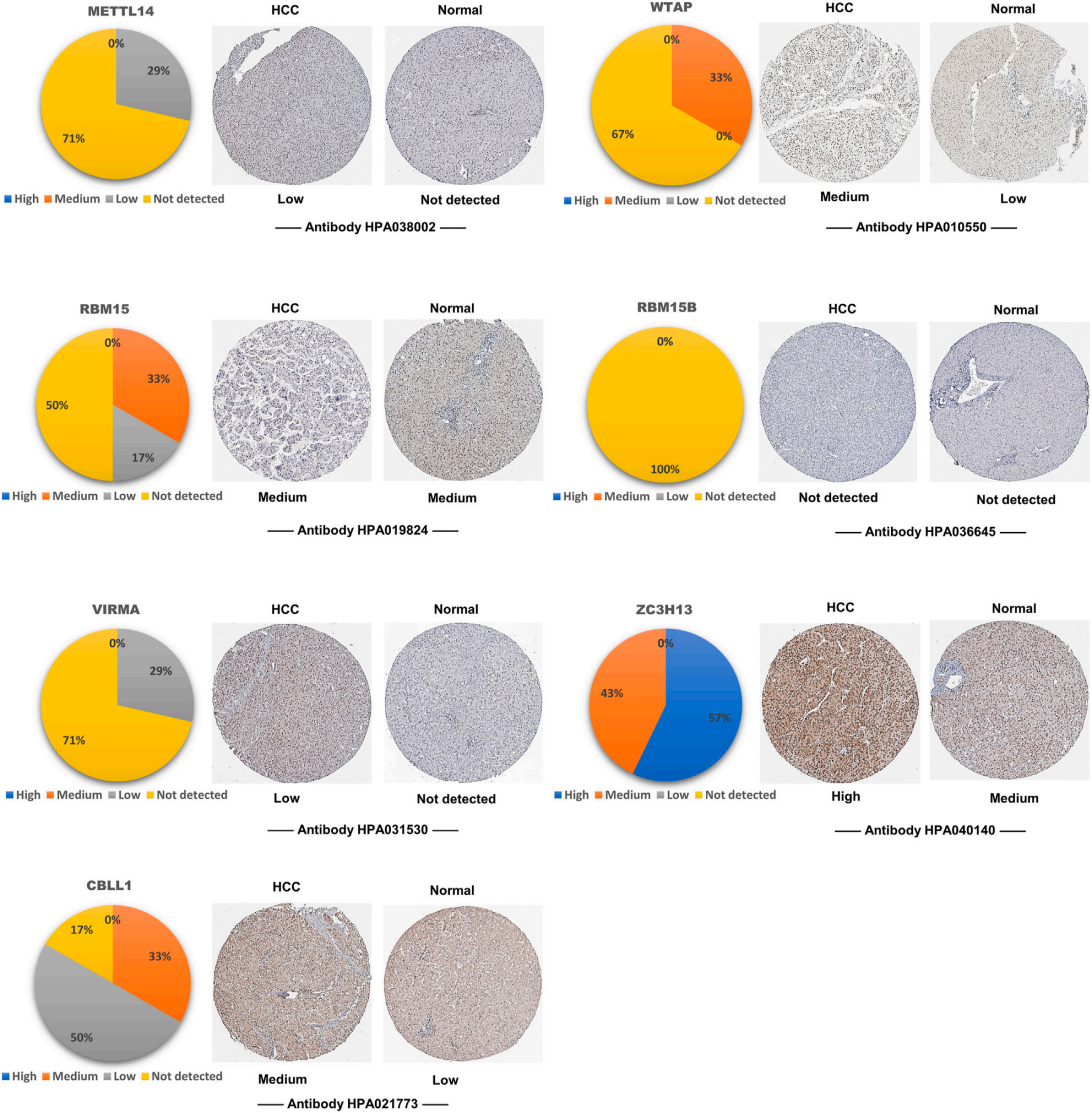
Tissue levels of the m^6^A writer complex in HCC (HPA). Representative immunohistochemistry (IHC) images of the m^6^A writer complex in HCC tissues. METTL3 data is temporarily missing in HPA database. The pie chart shows the proportion of IHC staining differences in HCC tissues. Only images with the most prominent tissue expression are shown.

To explore the expression levels of writer complex proteins in HCC tissues, we analyzed immunohistochemistry (IHC) data using the HPA database and found that except for the missing METTL3 data, other complex proteins displayed different extents of expression in HCC compared to normal tissues. Among them, expression of METTL14 and ZC3H13 proteins was not significantly increased, while expression of other components, especially WTAP, RBM15 and CBLL1 proteins, was increased to varying extents (Figure 3), which was basically consistent with their changes at the transcriptional level.

### Relationship Between the m^6^A Writer Complex and Clinicopathological Parameters in Hepatocellular Carcinoma

**FIGURE 4:**
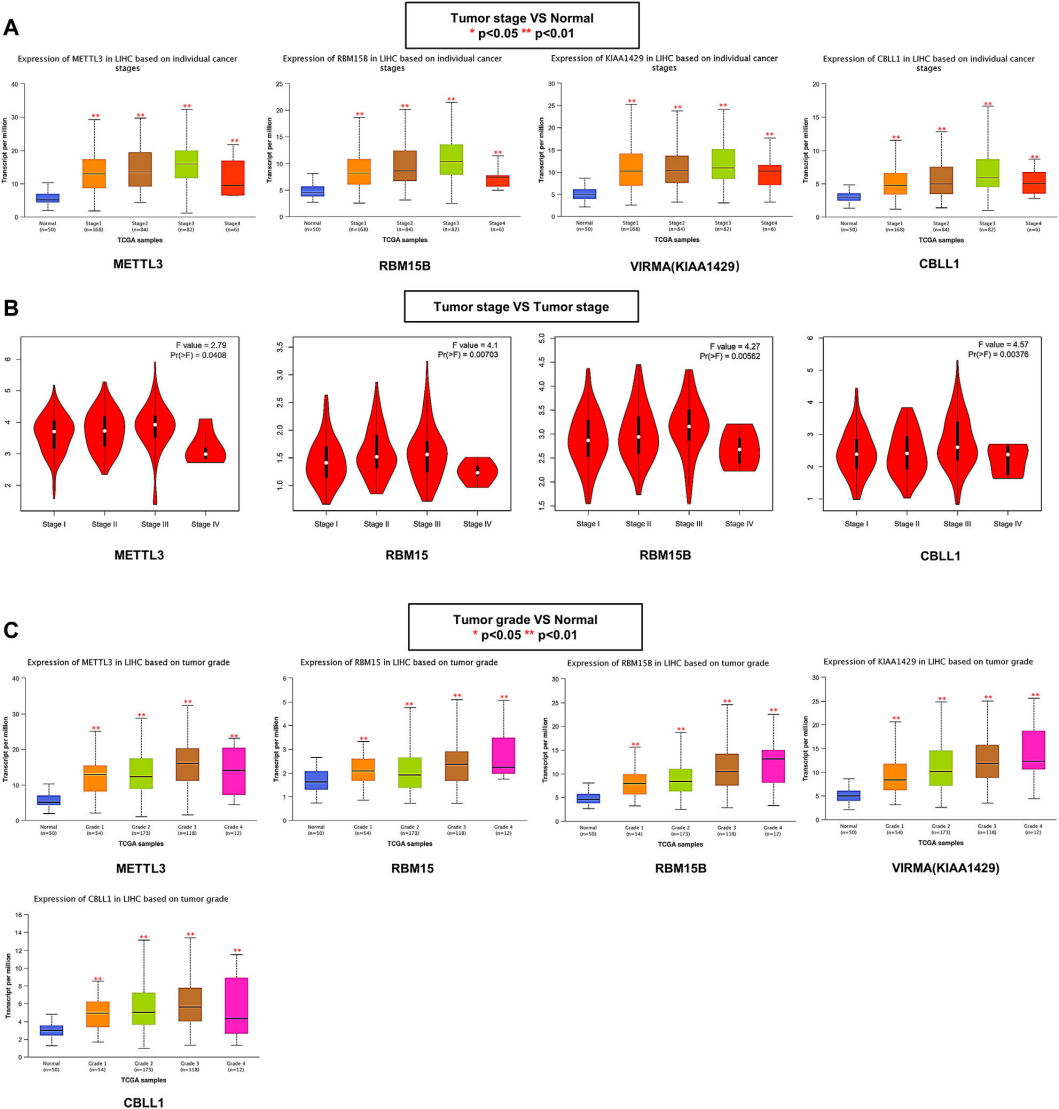
Relationship between the m^6^A writer complex and clinicopathological parameters in HCC. **(A)** Association of mRNA expression of the m^6^A writer complex with individual cancer stages in HCC (UALCAN). **(B)** The correlation between m^6^A writer complex expression and tumor stage in HCC (GEPIA). **(C)** Association of mRNA expression of the m^6^A writer complex with tumor grades in HCC (UALCAN). ANOVA, *p* < 0.05 was regarded as statistically significant. *p < 0.05, **p < 0.01, ***p < 0.001.

The association between the m^6^A writer complex and clinicopathological parameters was assessed based on an independent cohort of 424 patients with HCC from the TCGA database. Further analysis using the UALCAN database showed that the differences in writer complex expression between HCC and normal tissues might be related to tumor stage. The expression levels of METTL3, RBM15B, VIRMA, and CBLL1 in stages 1–4 were all significantly increased (ANOVA, *p* < 0.01) (Figure 4A), and expression levels of WTAP and RBM15 in stages 1–3 were significantly higher than those in adjacent tissues (ANOVA, *p* < 0.01), while expression of METTL14 and ZC3H13 was higher than that in adjacent tissues only in stage 3 (ANOVA, *p* < 0.05) (Supplementary Figure S1A). However, a stage plot from the GEPIA database (based on TCGA Project) showed that only expression of METTL3, RBM15, RBM15B and CBLL1 exhibited significant differences among HCC stages (ANOVA, *p* < 0.05) (Figure 4B; Supplementary Figure S1B). Furthermore, UALCAN revealed that the gene expression differences in METTL3, RBM15, RBM15B, VIRMA and CBLL1 may also be related to tumor grade (Figure 4C; Supplementary Figure S1C). The relationship between the m^6^A writer complex and other clinicopathological features was shown in Supplementary Table S1.

### Prognostic Value of the m^6^A Writer Complex in Hepatocellular Carcinoma

**FIGURE 5:**
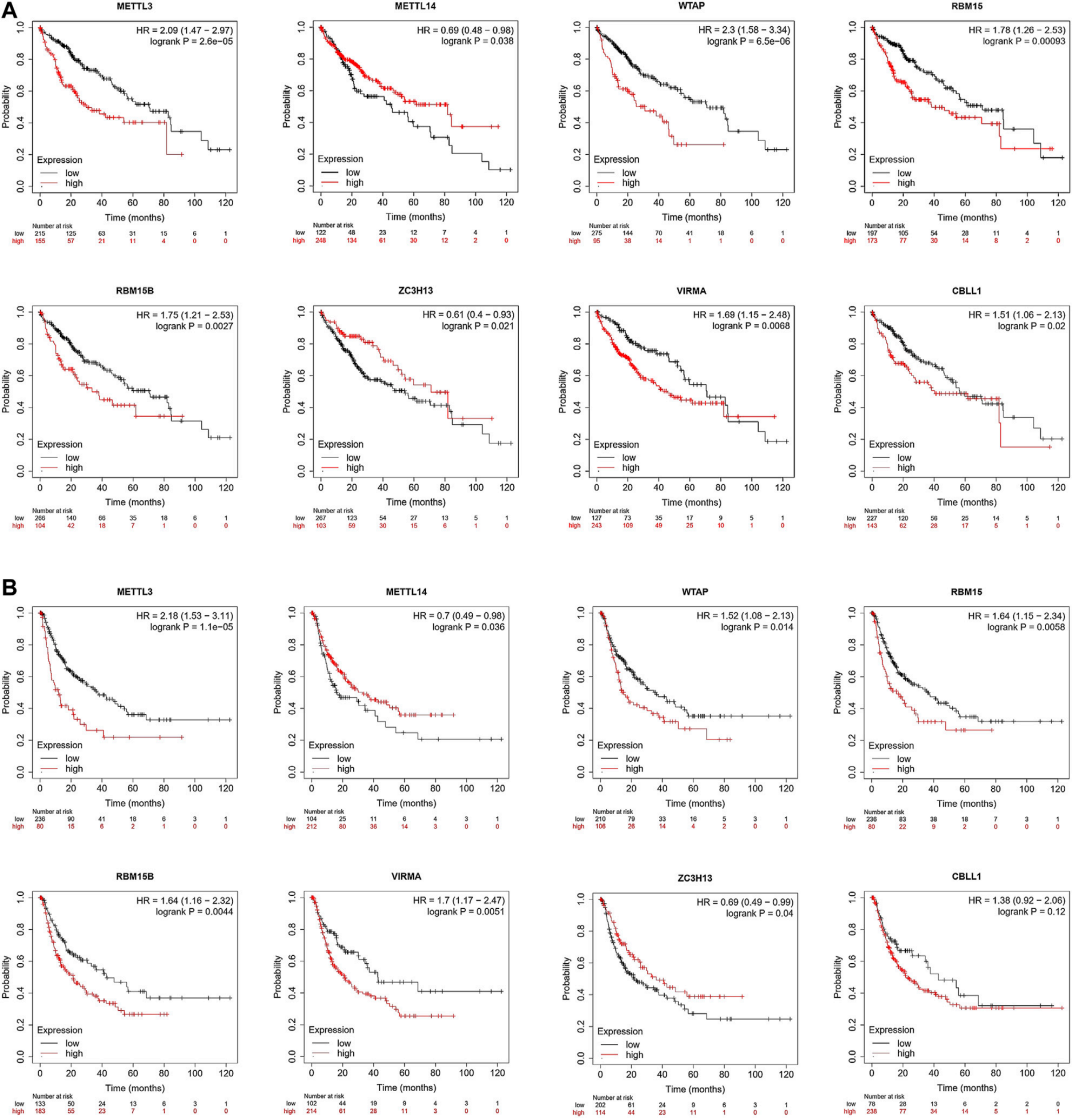
Prognostic value of the m^6^A writer complex in HCC (Kaplan–Meier Plotter). The correlation between expression of the m^6^A writer complex genes and overall survival (OS) **(A)** and relapse-free survival (RFS) **(B)** in HCC. Data are shown as the hazard ratio with a 95% confidence interval. Log-rank *p* < 0.05 was regarded as statistically significant.

Next, we used Kaplan–Meier Plotter tools to conduct survival analysis based on the TCGA database. The Kaplan–Meier curve and log-rank test analyses revealed that differences in the expression of the m^6^A writer complex significantly affected OS in patients with HCC (log-rank test, *p* < 0.05) (Figure 5A). Upregulated expression of METTL3, WTAP, RBM15, RBM15B, VIRMA, and CBLL1 and downregulated expression of METTL14 and ZC3H13 are markers of poor prognosis in HCC (Figure 5A), consistent with previous analytic results (Figure 2D). Moreover, the differential expression of other components of the writer complex was also significantly related to RFS in patients with HCC (log-rank test, *p* < 0.05), except for CBLL1 (Figure 5B), which may be related to the low number of HCC patients with CBLL1 upregulation in the TCGA database whose RFS was longer than 60 months, leading to statistical bias.

### Genetic Alterations Related to the m^6^A Writer Complex and Their Correlation With Clinicopathological Factors in Hepatocellular Carcinoma

**FIGURE 6:**
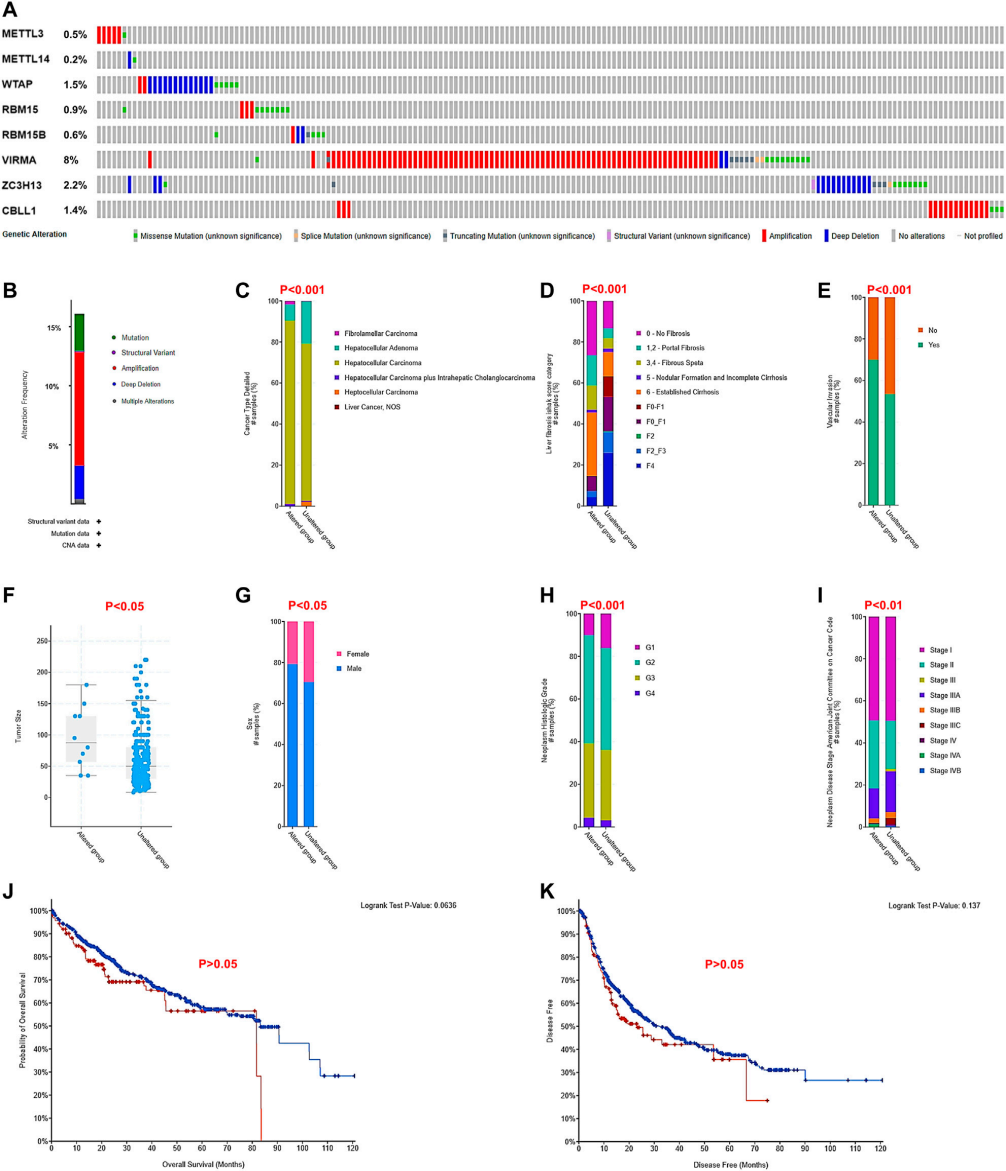
Genetic alterations related to the m^6^A writer complex and their correlation with clinicopathological factors in HCC (cBioPortal). **(A)** Amplification, deletion, and mutation of the m^6^A writer complex in HCC. **(B)** Genetic alteration summary of the m^6^A writer complex in HCC. The correlation between genetic alteration of the m^6^A writer complex and tumor type **(C)**, Ishak fibrosis score **(D)**, vascular invasion **(E)**, tumor volume **(F)**, sex **(G)**, tumor grade **(H)**, and tumor stage **(I)**. Chi-squared test, *p* < 0.05 was regarded as statistically significant. The relationship between genetic alteration of the m^6^A writer complex and OS **(J)** and DFS **(K)** of HCC patients. Log-rank *p* < 0.05 was regarded as statistically significant.

To explore the possible underlying mechanisms of differential expression of the m^6^A writer complex in HCC, we analyzed their gene alterations using the cBioPortal database. The results of the analysis revealed that two or more types of gene alterations were detected in 15% of cases (191/1267) (Figures 6A,B). Of these, amplification was more frequent in METTL3 (83%, 5/6), VIRMA (81%, 79/98) and CBLL1 (83%, 15/18), while WTAP (65%, 13/20), METTL14 (50%, 1/2) and ZC3H13 (50%, 14/28) were prone to deep deletions (Figure 6A). Further comparison with clinicopathological indicators revealed that altered the group was significantly associated with tumor type (Figure 6C), high Ishak fibrosis score (Figure 6D), high vascular invasion (Figure 6E), large tumor volume (Figure 6F) in male patients (Figure 6G) with high tumor grade (Figure 6H) and advanced tumor stage (Figure 6I) (Chi-Squared Test, *p* < 0.05). Unfortunately, the altered group did exhibit significantly altered OS (Figure 6J) or disease-free survival (DFS, Figure 6K) in patients with HCC (log-rank test, *p* > 0.05), which may be associated with their low mutation prevalence and multiple confounding factors.

### Coexpressed Genes and Interactions of the m^6^A Writer Complex in Hepatocellular Carcinoma

**FIGURE 7:**
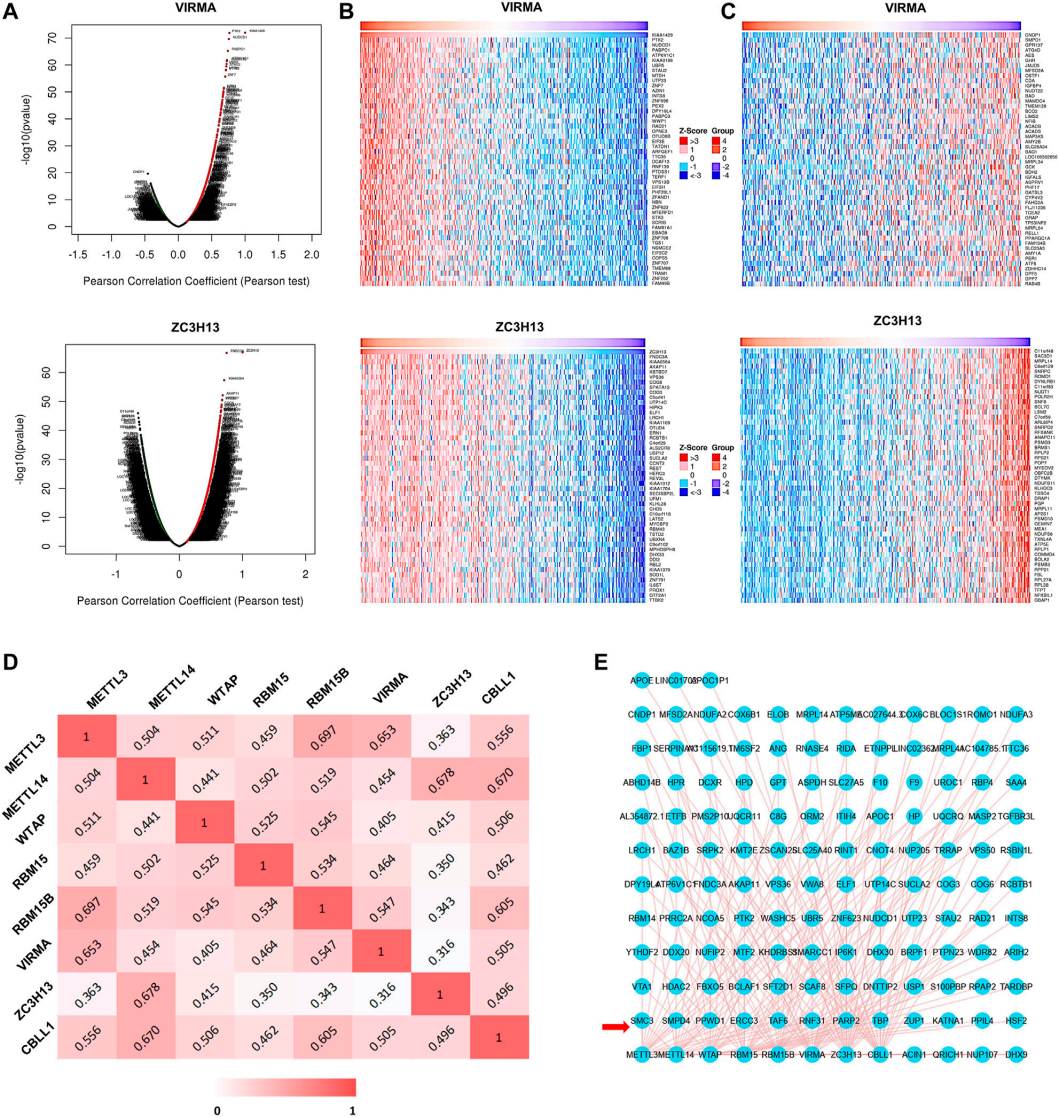
Coexpressed genes and interactions of the m^6^A writer complex in HCC. **(A)** Volcano plots show differentially expressed genes related to the m^6^A writer complex in HCC (LinkedOmics). Heatmaps show the top 50 genes positively **(B)** and negatively **(C)** correlated with the m^6^A writer complex in HCC (LinkedOmics). Red indicates positively correlated genes, and blue/green indicates negatively correlated genes. **(D)** Heatmap shows mRNA level correlation between the m^6^A writer-complex components based on Pearson correlation coefficient. **(E)** Network for the m^6^A writer complex and its 10 most frequently altered neighboring genes. The red arrow indicates the most frequently altered neighboring genes.

To explore the interaction between the m^6^A writer complex genes and coexpressed genes in HCC, we first used the LinkedOmics database to draw a volcano map of coexpressed genes related to the writer complex (Figure 7A; Supplementary Figure S2A). The top 50 positively (Figure 7B; Supplementary Figure S2B) and negatively (Figure 7C; Supplementary Figure S2C) regulated genes related to the writer complex are shown in the heatmap. We then applied the Timer database to analyze the Pearson correlation coefficient (r) between the complex components based on RNA-seq data and drew a heatmap (Figure 7D). The results showed that expression of each component was positively correlated (r > 0), of which the correlation between METTL3 and RBM15B was the strongest (r = 0.697), followed by METTL14 and ZC3H13 (r = 0.678) (Figure 7D). Next, we constructed a network of the m^6^A writer complex and its 10 most frequently altered neighboring genes using the igraph package and the ggraph package in R software. The network revealed several genes significantly associated with the m^6^A writer complex, including transcriptional regulators (TAF6, TBP), protein modification genes (PPWD1, RNF31) and DNA damage repair genes (PARP2, ERCC3) (Figure 7E).

### Protein–Protein Interactions and Functional Enrichment Analysis of the m^6^A Writer Complex in Hepatocellular Carcinoma

**FIGURE 8:**
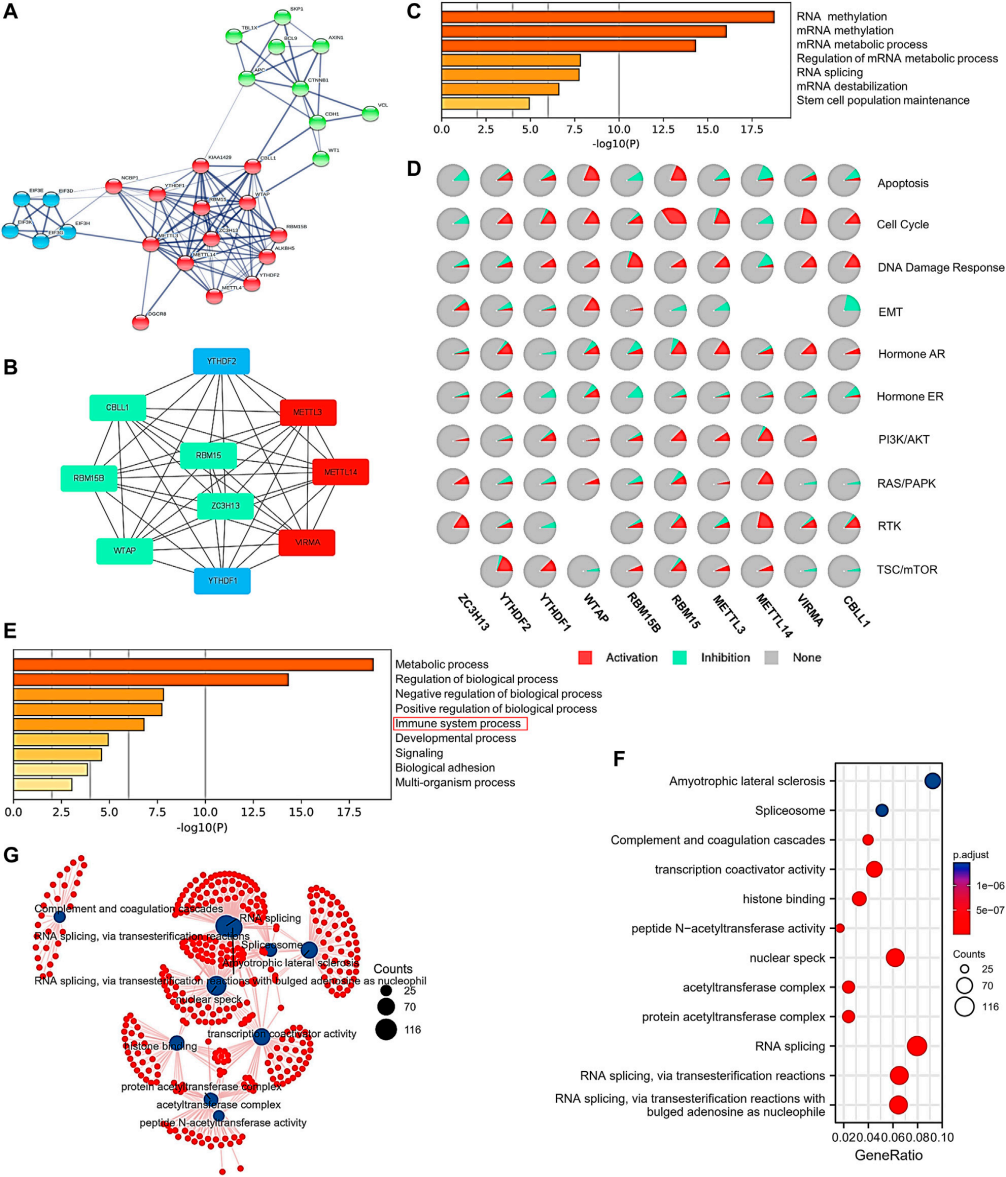
Protein–protein interactions (PPIs) and functional enrichment analysis of the m^6^A writer complex in HCC. **(A)** PPI network of the top 20 proteins related to the m^6^A writer complex in HCC (STRING). **(B)** Ten hub genes selected by Cytoscape from the PPI network. Red represents high degree value of the gene. Blue represents the hub genes except for the writer complex components. **(C)** Molecular function enrichment of the hub genes (Metascape). **(D)** Pathway enrichment of the hub genes (GSCALite). **(E)** Biological process enrichment of the hub genes (Metascape). **(F)** Gene Ontology (GO) and Kyoto Encyclopedia of Genes and Genomes (KEGG) pathway enrichment analysis of the writer complex and the top 200 coexpressed genes. **(G)** Network for GO and KEGG pathway enrichment.

To explore the interactions of protein expression between writer complex components in HCC, we used the STRING database to analyze the combined score of each component and create a heatmap (Supplementary Figure S3A). The results showed that WTAP protein expression displayed the strongest relationship with VIRMA, ZC3H13 and CBLL1 (combined score = 0. 999), followed by METTL3 versus METTL14 and WTAP (combined score = 0.998) and METTL14 versus WTAP (combined score = 0.998) (Supplementary Figure S3A). We then analyzed the protein–protein interaction (PPI) network associated with the m^6^A writer complex in HCC. We further used Cytoscape to map the hub gene network of the top 10 genes based on degree value rank (Figure 8A), and the results also showed that the protein interactions between the components of the writer complex were strong, especially between METTL3, METTL14 and VIRMA (Figure 8B). Notably, expression of the RNA-binding proteins YTHDF1 and YTHDF2 was also significantly associated with the m^6^A writer complex (Figure 8B), consistent with their combined functions in methylation regulation (Figure 1A). Next, we conducted GO and KEGG analyses using Metascape, GSCALite and the clusterProfiler R package to explore the specific function and biological pathways of the m^6^A writer complex identified in HCC. First, GO analysis of complex hub genes confirmed their ability to modify RNA methylation. Other functions may include RNA splicing, regulation of mRNA metabolism, and maintenance of stem cell function (Figure 8C). Pathway activity analysis suggested that the above functions were involved in activation of tumor-promoting pathways and/or inhibition of tumor-suppressing pathways, including apoptosis, cell cycle, DNA damage response and EMT (Figure 8D). Moreover, immune process regulation and signaling pathways were also involved, including AR/PR, PI3K/AKT, RAS/PAPK, RTK, and TSC/mTOR (Figure 8E; Supplementary Figure S3B). We then performed an extended GO and KEGG analysis by intersecting the top 200 coexpressed genes associated with each writer complex component. The results showed that the m6A writer complex may also involve histone binding, protein acetylation modification, transcription coactivator, and complement and coagulation cascades (Figure 8F; Supplementary Figure S3C). The function network is shown in Figure 8G.

### Correlations Between the m^6^A Writer Complex and Tumor Immunology in Hepatocellular Carcinoma

**FIGURE 9:**
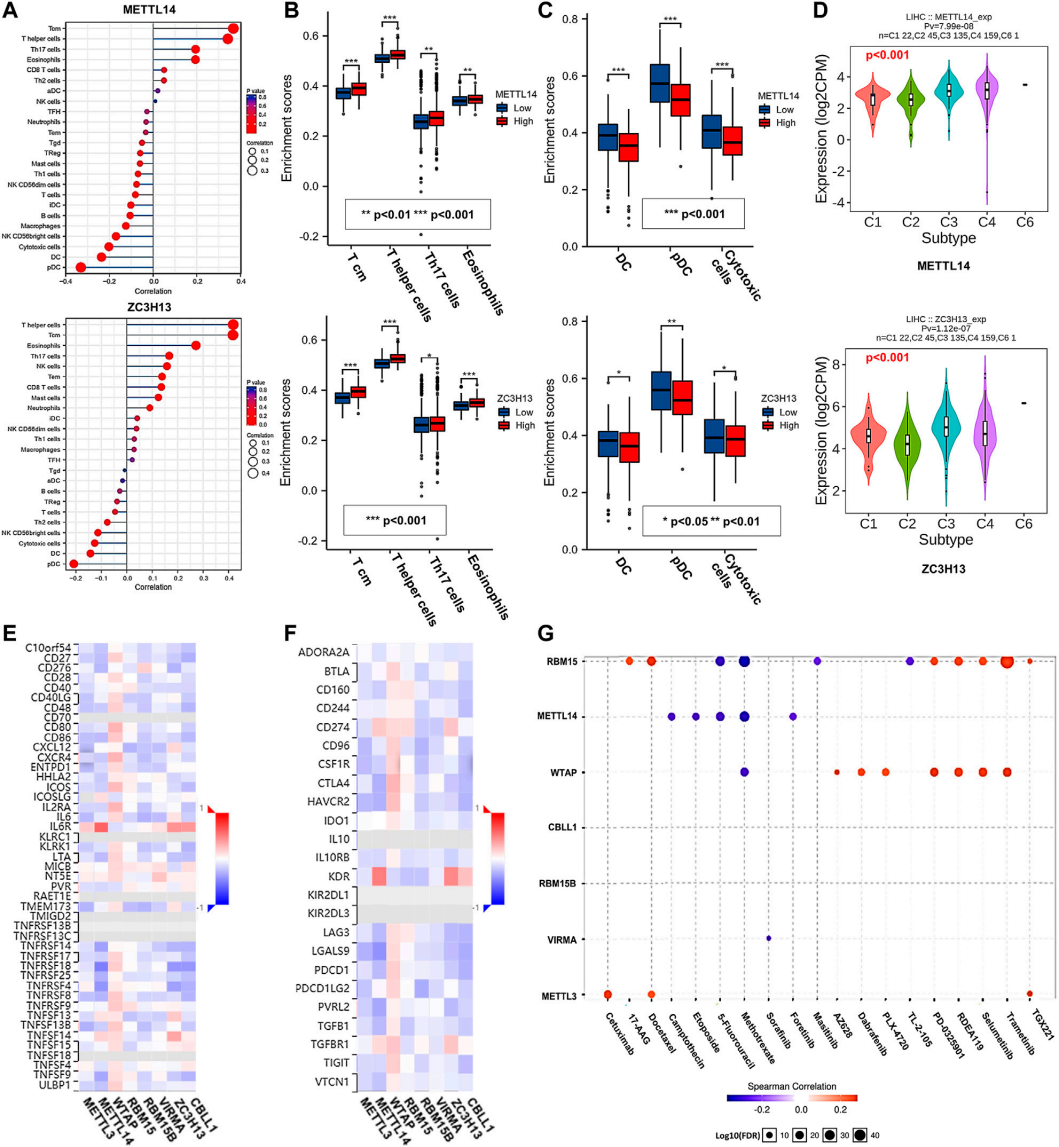
Correlations between the m^6^A writer complex and tumor immunology in HCC. **(A)** Relationship between the m^6^A writer complex and immune cell infiltration in HCC. Box plots show immune cells positively **(B)** and negatively **(C)** correlated with the m^6^A writer complex in HCC. **(D)** Correlations between the m^6^A writer complex and immune subtypes in HCC (TISIDB). Kruskal–Wallis test, *p* < 0.05 was regarded as statistically significant. C1: wound healing, C2: IFN-γ dominant, C3: inflammatory, C4: lymphocyte depleted, C5: immunologically quiet, C6: TGF-β dominant. Heatmaps show correlations between the m^6^A writer complex and the expression of immunostimulators **(E)** and immunoinhibitors **(F)** in HCC (TISIDB). **(G)** Correlations between the m^6^A writer complex and drug sensitivity in immune or targeted therapies (GSCALite). Red and blue represent positive and negative correlations, respectively.

Immune cell infiltration is an important part of the tumor microenvironment and is closely related to the development of cancer (Gajewski et al., 2013). Therefore, we applied the GSVA R package and TISIDB database to analyze the relationship between the m^6^A writer complex and immune cell infiltration in HCC (Figure 9A; Supplementary Figures S4A,B). Further intersection analysis of the results (Supplementary Table S2) revealed that METTL14 and ZC3H13 expression was positively correlated with Tcm, T helper cells, Th17 cells and eosinophil infiltration (Figure 9B). Expression of METTL3, WTAP, RBM15, RBM15B, VIRMA and CBLL1 was positively correlated with Tcm, T helper cell and Th2 cell infiltration (Supplementary Figure S4C). Interestingly, expression of all writer complex components was negatively correlated with the infiltration of pDCs, DCs and cytotoxic cells (Figure 9C; Supplementary Figure S4D). Furthermore, expression of the m^6^A writer complex was significantly correlated with the immune subtypes of HCC (Kruskal–Wallis test, *p* < 0.05) (Figure 9D; Supplementary Figure S4E). Next, we analyzed the correlation between the m^6^A writer complex and immunostimulators (Figure 9E), immunoinhibitors (Figure 9F), MHC molecules (Supplementary Figure S4F), chemokines (Supplementary Figure S4G) and chemokine receptors (Supplementary Figure S4H) of infiltrating immune cells in HCC. These results could provide important information for predicting potential therapeutic targets. Finally, we used the GSCALite online tool based on the GDSC database to analyze the relationship between the m^6^A writer complex and drug sensitivity in immune or targeted therapies (Figure 9G). The results revealed that METTL3, WTAP and RBM15 expression was positively correlated with the sensitivity of several immune or targeted drugs, which might be potential biomarkers for drug screening.

## Discussion

Previous studies have reported that m^6^A regulators are dysregulated in many cancers, including HCC, and may have prognostic value (8–10). However, the m^6^A writer complex, which are responsible for m^6^A installation, has not been independently examined, and a specific bioinformatics analysis of this complex has not yet been performed. In this study, we used multiple public databases to reveal that expression levels of METTL3, VIRMA and CBLL1 were significantly increased in the m^6^A writer complex, while expression levels of METTL14 and ZC3H13 were significantly decreased in HCC, which was closely related to clinicopathological factors, such as tumor stage and prognosis, confirming their potential role as biomarkers for diagnosis and prognosis determination. In addition, we explored the possible underlying mechanisms of the m^6^A writer complex involved in the carcinogenesis and progression of HCC and its relationship with the tumor immune response. This may provide potential targets for treatment with clinical translational implications.

METTL3 is an S-adenosylmethionine (SAM)-binding protein that is the only component in the m^6^A writer complex with methyltransferase catalytic activity (Śledź and Jinek, 2016). Recent studies have shown that METTL3 is upregulated and associated with poor prognosis in gastrointestinal malignancies, including liver cancer, pancreatic cancer and colorectal cancer (Li et al., 2019). Subsequent *in vitro* and *in vivo* experiments have confirmed that downregulation of METTL3 inhibits tumor growth and metastasis (Li et al., 2019). Mechanistically, METTL3 increases methylation levels of suppressor of cytokine signaling 2 (SOCS2) mRNA, promotes its degradation through a m^6^A-YTHDF2-dependent mechanism, and inhibits SOCS2 expression in HCC tissues, thereby promoting HCC progression (Chen et al., 2018). Moreover, METTL3 also accelerates HCC progression by methylating the transcription factor Snail of EMT and promoting its translation, a process that may involve the interaction between YTHDF1 and eukaryotic translation elongation factor 2 (eEF-2) (Lin et al., 2019). Therefore, METTL3 overexpression in HCC promotes the development of HCC by binding to the m^6^A reader proteins YTHDF1 and YTHDF2 and subsequently regulating downstream signaling pathways, which is consistent with the finding that YTHDF1 and YTHDF2 are hub genes in this study. Further GO and KEGG analyses also confirmed the key biological processes involved, such as anti-apoptosis, promotion of proliferation, and EMT, suggesting that the writer complex plays a key role in regulating HCC cell proliferation and inducing chemotherapy resistance. Notably, the co-expressed gene network revealed that the expression of transcriptional regulatory factor (TBP) and DNA damage repair genes (PARP2, ERCC3) may also be significantly correlated with the m^6^A writer complex in HCC, and recent studies have provided more evidence. A very recent study showed that TATA-binding protein (TBP) can positively regulate METTL3 transcription, which further upregulates PDK4 expression in HCC cells (Li et al., 2020b). PDK4 is one of the key factors involved in the regulation of glycolysis in cancer cells, which can promote tumor metabolic remodeling and contribute to chemoresistance (Li et al., 2020b). Therefore, the TBP/METTL3/PDK4 axis may be a novel mechanism involved in HCC progression. However, the specific mechanisms by which transcription factors regulate the m^6^A writer complex remain to be further explored. Additionally, METTL3 can recruit the key DNA polymerase κ (Pol κ) to DNA damage sites through the PARP/METTL3/Pol κ axis, promoting ultraviolet (UV)-induced DNA damage repair and cell survival (Xiang et al., 2017). Moreover, METTL3-mediated upregulation of yes-associated protein (YAP) leads to DNA damage repair by upregulating the expression of downstream excision repair cross-complementing 1 (ERCC1) in NSCLC (Jin et al., 2019). DNA damage repair is one of the key mechanisms for cancer cells to survive chemotherapy. Therefore, METTL3-mediated recruitment or expression of key enzymes in DNA damage repair may facilitate tumor progression and chemoresistance in HCC. METTL14, an allosteric adapter of METTL3, forms a heterodimer with METTL3 to stabilize the writer complex and recruit substrate RNA ((Śledź and Jinek, 2016), (Wang et al., 2016)). The crystal structure and biochemical evidence suggested that METTL3, rather than METTL14, is the unique catalytic subunit (Wang et al., 2016). The different roles of METTL14 and METTL3 in methylation may underlie their conflicting expression changes in HCC. In addition, METTL3, but not METTL14, exerts the methyltransferase independent function to potentiate mRNA translation, which might also contribute to their divergent expression and biological function ((Lin et al., 2016), (Choe et al., 2018)). Our study confirmed reports that METTL14 acts as a tumor suppressor in HCC. *In vitro* experiments also demonstrated that METTL14 knockdown promotes tumor cell proliferation and invasion by activating PI3K/Akt signaling (Zhang et al., 2019), which is consistent with our pathway enrichment results. Analogous to METTL3, METTL14 combined with YTHDF1 can bind to the DNA damage-binding protein 2 (DDB2) transcript, regulating DDB2 m^6^A methylation and translation, promoting UV-induced DNA damage repair and suppressing skin tumorigenesis (Yang et al., 2021). Consequently, these data suggest that METTL3 and METTL14 may serve as potential therapeutic targets and facilitate the development of new strategies to sensitize cancer cells to DNA-damaging agents in HCC. Interestingly, although METTL3 and METTL14 seem to play completely opposite roles in HCC progression, which may not be the case in other tumors, such as downregulated METTL3 expression detected in approximately 70% of endometrial cancers, which may be related to tumor heterogeneity (Liu et al., 2018a). VIRMA (also known as KIAA1429) interacts with WTAP to direct the writer complex to regionally selective methylation (Yue et al., 2018), and it is upregulated in HCC with poor prognosis (Lan et al., 2019), which is consistent with our study. Mechanistically, GATA3 is a direct downstream target of VIRMA-induced m^6^A methylation modification, which leads to downregulation of GATA3 mRNA expression and promotes invasion and migration of HCC cells (Lan et al., 2019). CBLL1, or HAKAI, is a class of E3 ubiquitin ligases that interacts with E-cadherin (Fujita et al., 2002). CBLL1 has been reported to be overexpressed and associated with poor prognosis in non-small cell lung cancer (NSCLC) and esophageal cancer (EC) (Weng et al., 2019; Zhao et al., 2021). Recent studies have shown that CBLL1 interacts with E-cadherin phosphorylated by Src kinase to induce ubiquitination and endocytosis of E-cadherin in HCC, which is associated with the transformation of aggressive phenotypes of tumor cells (Lu et al., 2018). Targeted knockdown of CBLL1 inhibits the growth of tumor cells (Liu et al., 2018c), which may be the potential mechanism of CBLL1 overexpression that is related to poor prognosis in this study. ZC3H13 is a prototypical CCCH-type zinc finger protein that binds to RBM15/RBM15B and attaches to WTAP in the m^6^A writer complex to improve catalytic potency (Zaccara et al., 2019). In contrast to its upregulation in cholangiocarcinoma and EC (Guo et al., 2021), we found that ZC3H13 acts as a tumor suppressor in HCC, consistent with the findings in breast and ovarian cancer (Zhang et al., 2020b; Wang et al., 2021a), suggesting functional diversity of ZC3H13 in different tumors. Very recently, an independent study indicated that ZC3H13 suppressed the progression of HCC through m^6^A-PKM2-mediated glycolysis and sensitized HCC cells to cisplatin, which offered a novel insight into ZC3H13 downregulation in HCC (Wang et al., 2021b). Moreover, another study on colorectal cancer found that ZC3H13 inhibits tumor cell proliferation and invasion by downregulating the expression of Snail, cyclin D1 and cyclin E1 by inhibiting the RAS signaling pathway (Zhu et al., 2019). In addition, ZC3H13 levels are also positively correlated with ER and PR expression in breast cancer (Zhang et al., 2020b). These findings might explain our functional enrichment results. In addition, Wilms’ tumor-associated protein acts as a key METTL3 adaptor and interacts with other components of the writer complex to participate in specific m^6^A methylation modification (Ping et al., 2014). However, the carcinogenic roles of WTAP and RBM15/15B in HCC remain controversial. For example, the study of Ma et al.(Ma et al., 2017) did not show that WTAP was overexpressed in HCC, but Chen et al. (Chen et al., 2019) found that WTAP expression was upregulated and promoted HCC progression through the HuR-ETS1-p21/p27 axis. These seemingly contradictory conclusions may be related to the adaptive stress of the m^6^A writer complex in HCC.

Given the important role of intratumoral immune cells, we also evaluated the correlation between the m^6^A writer complex and immune cell infiltration in HCC. Notably, expression of the tumor suppressors METTL14 and ZC3H13 was positively correlated with the infiltration of Tcm cells, Th17 cells, and eosinophils, consistent with previous findings that these cells are associated with a favorable prognosis of malignant tumors (Cua and Tato, 2010; Steel et al., 2010; Xu et al., 2019). Paradoxically, we found that tumor suppressors were negatively correlated with the infiltration of pDCs, DCs, and cytotoxic cells, and the tumor promoters METTL3, VIRMA, and CBLL1 were positively correlated with Tcm and Th2 cell infiltration. Infiltration of DCs, Tcm and their derived cytotoxic T cells, along with Th2 cells, are generally considered to be protective factors for HCC (Foerster et al., 2018; Lawal et al., 2021), which may be related to changes in the balance between DC subsets or T effector cells and regulatory T cells in tumors (Lawal et al., 2021; Gao et al., 2007), suggesting that stratification of immune cell infiltration is the key to achieving effective treatments. Therefore, the interplay between the m^6^A writer complex and tumor microenvironment may be an important mechanism for the tumorigenesis and progression of HCC. However, more specific mechanisms remain to be clarified.

## Conclusion

In conclusion, our study systematically illustrated the expression changes and prognostic value of the m^6^A writer complex in HCC. Expression of several specific complex components correlates with pathways involved in carcinogenesis, tumor development, and tumor metastasis. Furthermore, the m^6^A writer complex may be involved in the regulation of immune cell infiltration and immune targets. Therefore, our findings may help to provide new insights available to improve the diagnosis, improve treatment design, and ultimately improve the prognosis of HCC. However, further experimental studies are needed to confirm these conclusions.

## Data Availability

The original contributions presented in the study are included in the article/Supplementary Material, further inquiries can be directed to the corresponding author.
