# Supplementary figures and images for: Diagnostic, Therapeutic, and Prognostic Value of the m6A Writer Complex in Hepatocellular Carcinoma
Source: Front Cell Dev Biol. 2022 Feb 9;10:822011. doi: 10.3389/fcell.2022.822011 (PMC8864226; doi:10.3389/fcell.2022.822011)

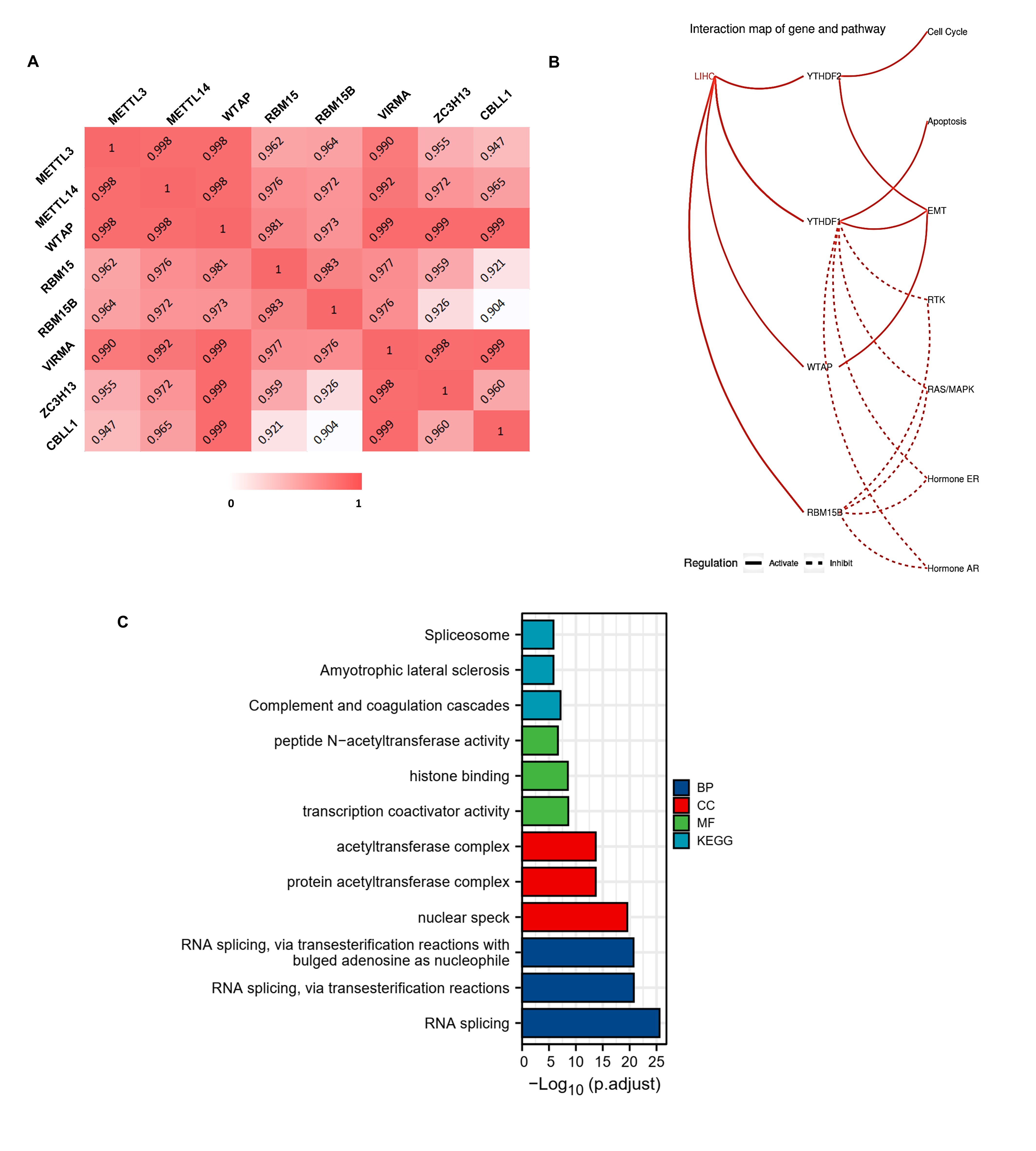

Supplement: Supplementary file 1 [file Image3.JPEG]

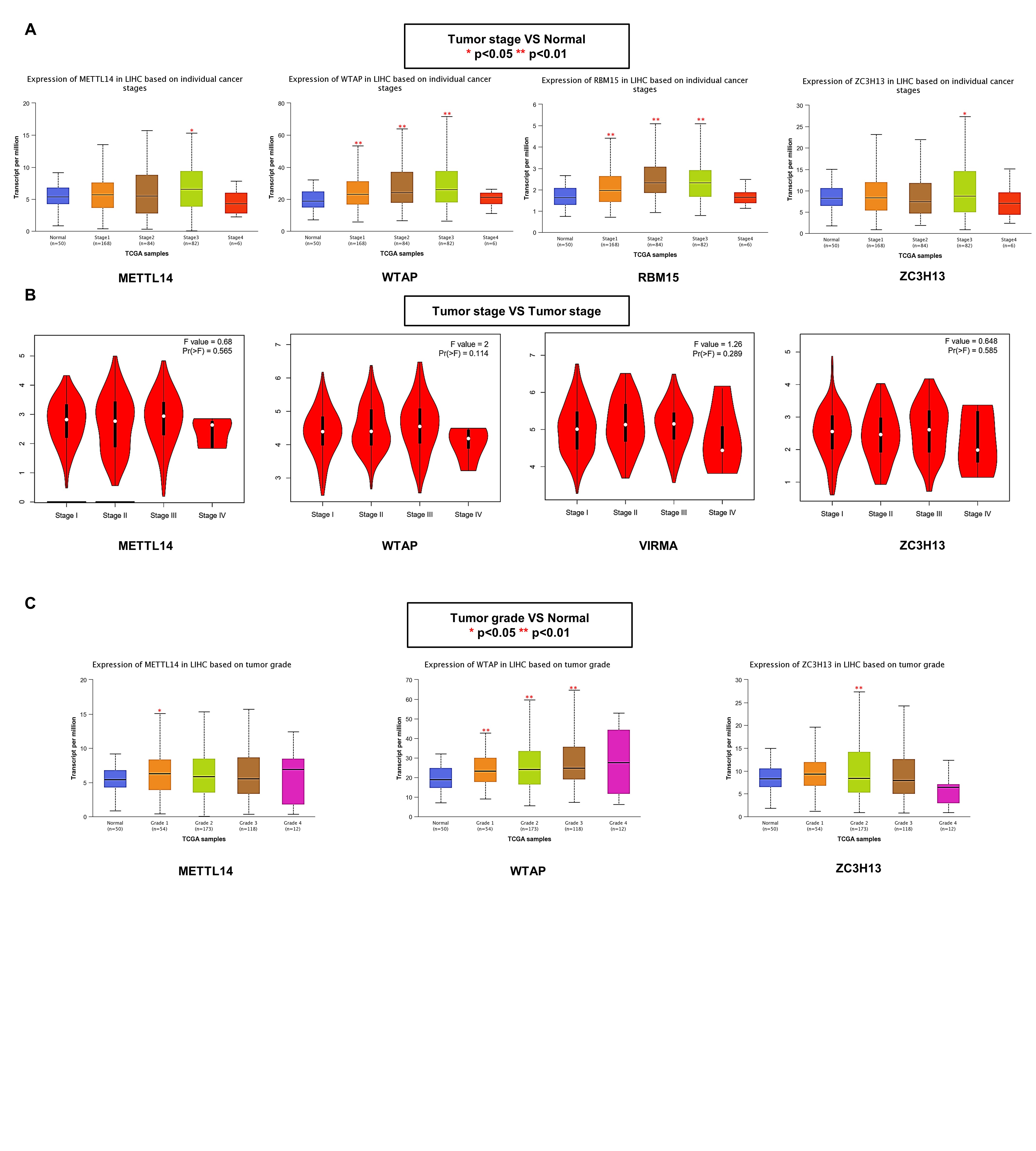

Supplement: Supplementary file 3 [file Image1.JPEG]

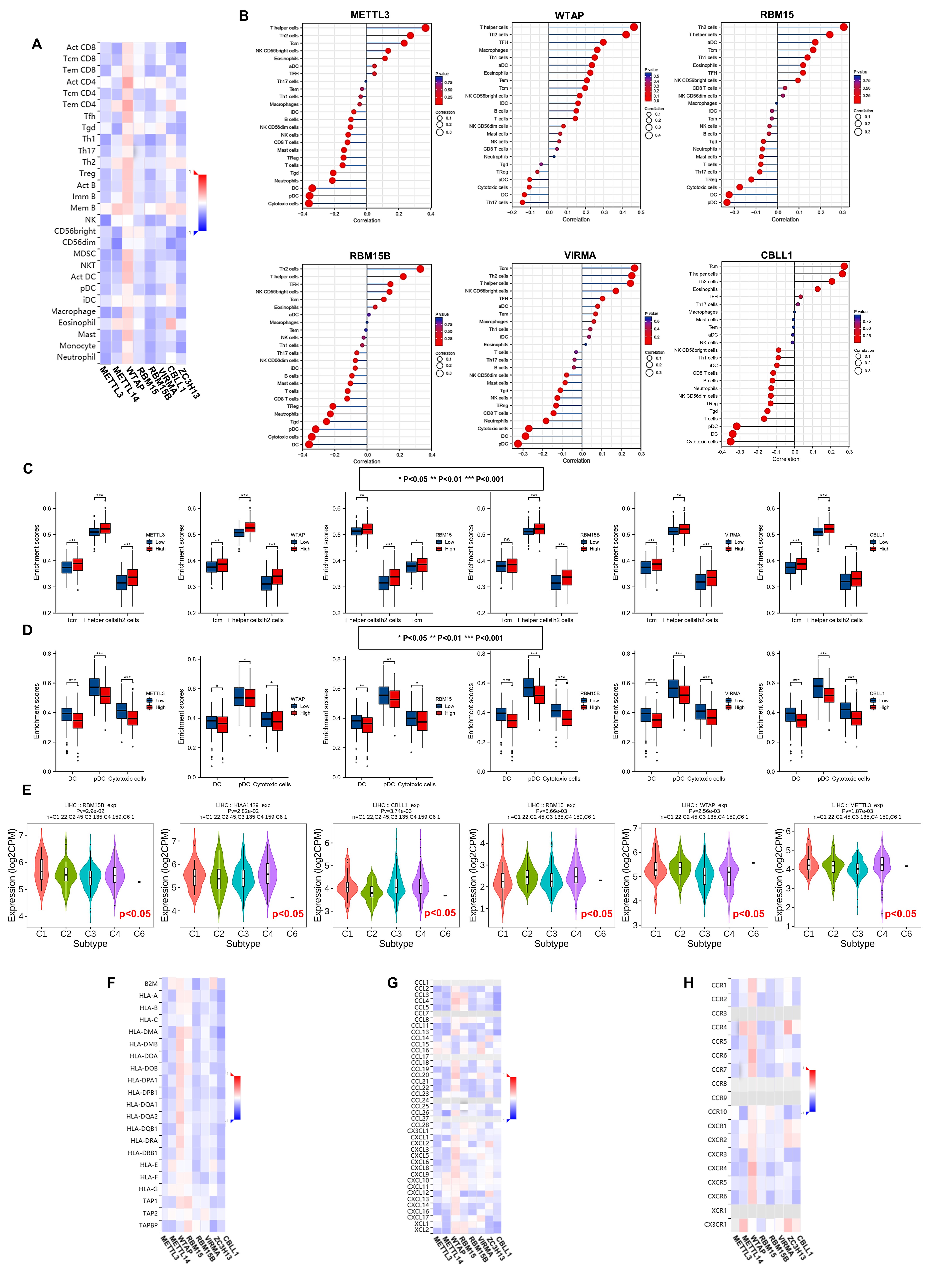

Supplement: Supplementary file 4 [file Image4.JPEG]

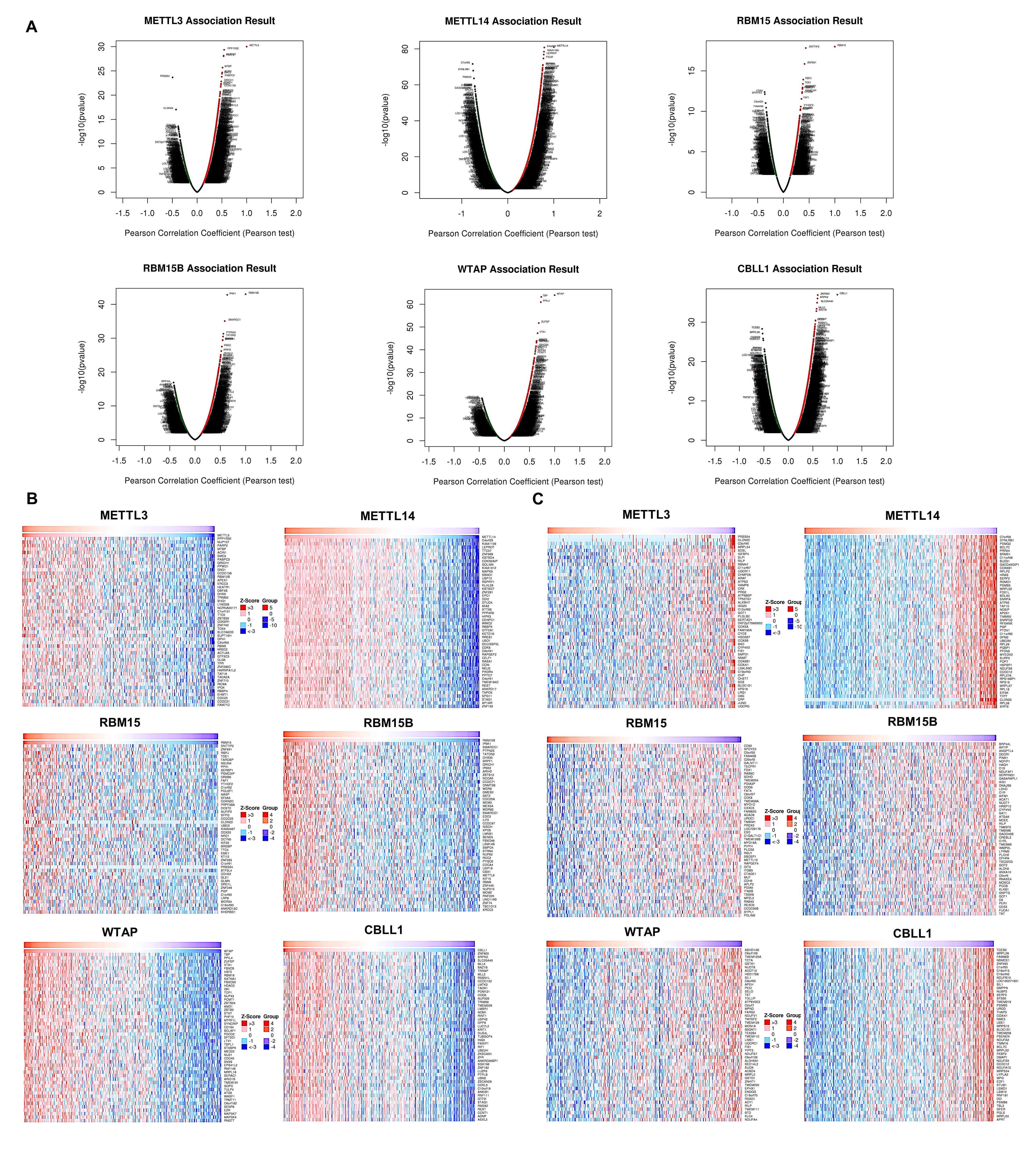

Supplement: Supplementary file 5 [file Image2.JPEG]
