# Supplementary material for: Diagnostic, Therapeutic, and Prognostic Value of the m6A Writer Complex in Hepatocellular Carcinoma
Source: Front Cell Dev Biol. 2022 Feb 9;10:822011. doi: 10.3389/fcell.2022.822011 (PMC8864226; doi:10.3389/fcell.2022.822011)
Supplement: Supplementary file 2 [file Table1.DOCX]

| **Characteristics** | **METTL3** | | **METTL14** | | **WTAP** | | **RBM15** | | **RBM15B** | | **VIRMA** | | **ZC3H13** | | **CBLL1** | |
| --- | --- | --- | --- | --- | --- | --- | --- | --- | --- | --- | --- | --- | --- | --- | --- | --- |
|  | **OR** | ***P*** | **OR** | ***P*** | **OR** | ***P*** | **OR** | ***P*** | **OR** | ***P*** | **OR** | ***P*** | **OR** | ***P*** | **OR** | ***P*** |
| **Age (>60 vs. <=60)** | **0.816 (0.542-1.225)** | **0.326** | **1.310 (0.872-1.971)** | **0.194** | **0.748 (0.497-1.124)** | **0.163** | **0.748 (0.497-1.124)** | **0.163** | **0.816 (0.542-1.225)** | **0.326** | **0.493 (0.325-0.744)** | **<0.001** | **1.078 (0.718-1.619)** | **0.719** | **0.764 (0.507-1.147)** | **0.194** |
| **Gender (Female vs. Male)** | **0.929 (0.602-1.434)** | **0.740** | **1.375 (0.891-2.130)** | **0.151** | **1.246 (0.808-1.927)** | **0.320** | **1.076 (0.698-1.661)** | **0.740** | **1.375 (0.891-2.130)** | **0.151** | **0.539 (0.346-0.835)** | **0.006** | **1.025 (0.664-1.582)** | **0.912** | **0.976 (0.632-1.506)** | **0.912** |
| **BMI (>25 vs. <=25)** | **0.670 (0.435-1.028)** | **0.068** | **1.056 (0.688-1.620)** | **0.804** | **0.855 (0.557-1.312)** | **0.473** | **0.743 (0.483-1.140)** | **0.174** | **0.581 (0.376-0.893)** | **0.014** | **0.758 (0.493-1.163)** | **0.205** | **1.188 (0.774-1.825)** | **0.431** | **0.689 (0.447-1.057)** | **0.089** |
| **AFP (ng/ml) (>400 vs. <=400)** | **2.307 (1.308-4.151)** | **0.004** | **1.035 (0.593-1.805)** | **0.903** | **1.548 (0.887-2.726)** | **0.126** | **0.938 (0.536-1.634)** | **0.820** | **3.562 (1.982-6.614)** | **<0.001** | **1.840 (1.050-3.275)** | **0.035** | **0.673 (0.380-1.177)** | **0.168** | **1.309 (0.751-2.290)** | **0.342** |
| **Albumin(g/dl) (>=3.5 vs. <3.5)** | **1.134 (0.662-1.954)** | **0.648** | **1.082 (0.631-1.858)** | **0.775** | **1.298 (0.756-2.249)** | **0.346** | **1.088 (0.635-1.872)** | **0.758** | **0.941 (0.549-1.617)** | **0.826** | **1.596 (0.928-2.785)** | **0.094** | **0.952 (0.555-1.634)** | **0.858** | **1.478 (0.856-2.588)** | **0.165** |
| **Prothrombin time (s) (>4 vs. <=4)** | **0.698 (0.421-1.150)** | **0.160** | **1.133 (0.690-1.866)** | **0.621** | **0.848 (0.514-1.395)** | **0.518** | **0.582 (0.350-0.960)** | **0.035** | **0.739 (0.446-1.218)** | **0.238** | **0.624 (0.374-1.029)** | **0.067** | **1.478 (0.898-2.448)** | **0.126** | **0.940 (0.570-1.546)** | **0.809** |
| **T stage (T3&T4 vs. T1&T2)** | **1.343 (0.839-2.162)** | **0.220** | **1.114 (0.696-1.785)** | **0.653** | **1.424 (0.889-2.295)** | **0.143** | **1.383 (0.864-2.225)** | **0.179** | **2.093 (1.297-3.421)** | **0.003** | **1.286 (0.804-2.067)** | **0.295** | **0.993 (0.620-1.589)** | **0.976** | **1.601 (0.998-2.589)** | **0.053** |
| **N stage (N1 vs. N0)** | **2.687 (0.339-54.709)** | **0.395** | **3.246 (0.409-66.099)** | **0.311** | **2.817 (0.355-57.360)** | **0.373** | **0.939 (0.111-7.923)** | **0.950** | **2.953 (0.373-60.136)** | **0.351** | **0.854 (0.101-7.207)** | **0.876** | **74931230.840 (0.000-NA)** | **0.994** | **2.562 (0.323-52.174)** | **0.418** |
| **M stage (M1 vs. M0)** | **0.314 (0.015-2.487)** | **0.318** | **0.376 (0.018-2.976)** | **0.399** | **0.359 (0.018-2.845)** | **0.378** | **0.333 (0.016-2.640)** | **0.344** | **0.314 (0.015-2.487)** | **0.318** | **0.985 (0.117-8.308)** | **0.988** | **0.338 (0.017-2.680)** | **0.351** | **0.314 (0.015-2.487)** | **0.318** |
| **Adjacent hepatic tissue inflammation (Mild&Severe vs. None)** | **1.818 (1.088-3.058)** | **0.023** | **0.830 (0.498-1.382)** | **0.474** | **1.643 (0.984-2.760)** | **0.059** | **1.289 (0.774-2.151)** | **0.330** | **1.169 (0.700-1.955)** | **0.551** | **1.336 (0.802-2.234)** | **0.267** | **1.125 (0.675-1.878)** | **0.652** | **1.019 (0.611-1.699)** | **0.944** |
| **Child-Pugh grade (B&C vs. A)** | **1.168 (0.481-2.838)** | **0.728** | **2.413 (0.977-6.534)** | **0.065** | **0.555 (0.205-1.372)** | **0.218** | **0.507 (0.187-1.251)** | **0.154** | **1.482 (0.614-3.649)** | **0.381** | **1.568 (0.650-3.944)** | **0.322** | **2.327 (0.956-6.039)** | **0.069** | **2.242 (0.922-5.818)** | **0.082** |
| **Fibrosis ishak score (3/4&5/6 vs. 0&1/2)** | **1.876 (1.093-3.248)** | **0.023** | **1.426 (0.835-2.446)** | **0.195** | **1.340 (0.781-2.309)** | **0.289** | **1.374 (0.805-2.356)** | **0.246** | **1.192 (0.696-2.046)** | **0.523** | **1.025 (0.598-1.757)** | **0.928** | **1.019 (0.596-1.740)** | **0.946** | **1.738 (1.013-3.002)** | **0.046** |
| **Vascular invasion (Yes vs. No)** | **1.258 (0.792-2.001)** | **0.332** | **1.301 (0.819-2.071)** | **0.265** | **1.231 (0.775-1.958)** | **0.379** | **1.536 (0.966-2.454)** | **0.070** | **1.106 (0.696-1.758)** | **0.670** | **1.455 (0.916-2.321)** | **0.113** | **1.019 (0.642-1.619)** | **0.935** | **0.916 (0.575-1.455)** | **0.709** |
