# Supplementary material for: Diagnostic, Therapeutic, and Prognostic Value of the m6A Writer Complex in Hepatocellular Carcinoma
Source: Front Cell Dev Biol. 2022 Feb 9;10:822011. doi: 10.3389/fcell.2022.822011 (PMC8864226; doi:10.3389/fcell.2022.822011)
Supplement: Supplementary file 6 [file Table2.DOCX]

| Gene | Correlation | Immune cells (p<0.05) | | Correlation coefficient (Pearson) | | P value (Pearson) |
| --- | --- | --- | --- | --- | --- | --- |
| ZC3H13 | **Positive** | **Th17 cells** | 0.144 | | 0.005 | |
|  |  | **Tem** | 0.121 | | 0.019 | |
|  |  | **Tcm** | 0.427 | | <0.001 | |
|  |  | **T helper cells** | 0.432 | | <0.001 | |
|  |  | **NK cells** | 0.165 | | 0.001 | |
|  |  | **Eosinophils** | 0.263 | | <0.001 | |
|  |  | **CD8 T cells** | 0.112 | | 0.030 | |
|  | **Negative** | **pDC** | -0.226 | | <0.001 | |
|  |  | **DC** | -0.133 | | 0.010 | |
|  |  | **Cytotoxic cells** | -0.137 | | 0.008 | |
| WTAP | **Positive** | **Th2 cells** | 0.440 | | <0.001 | |
|  |  | **Th1 cells** | 0.251 | | <0.001 | |
|  |  | **TFH** | 0.313 | | <0.001 | |
|  |  | **Tem** | 0.216 | | <0.001 | |
|  |  | **Tcm** | 0.211 | | <0.001 | |
|  |  | **T helper cells** | 0.511 | | <0.001 | |
|  |  | **T cells** | 0.169 | | 0.001 | |
|  |  | **NK CD56bright cells** | 0.161 | | 0.002 | |
|  |  | **Macrophages** | 0.290 | | <0.001 | |
|  |  | **iDC** | 0.181 | | <0.001 | |
|  |  | **Eosinophils** | 0.221 | | <0.001 | |
|  |  | **B cells** | 0.169 | | 0.001 | |
|  |  | **aDC** | 0.235 | | <0.001 | |
|  | **Negative** | **Th17 cells** | -0.133 | | 0.010 | |
|  |  | **pDC** | -0.147 | | 0.004 | |
|  |  | **DC** | -0.142 | | 0.006 | |
|  |  | **Cytotoxic cells** | -0.116 | | 0.025 | |
| VIRMA | **Positive** | **Th2 cells** | 0.264 | | <0.001 | |
|  |  | **Tcm** | 0.281 | | <0.001 | |
|  |  | **T helper cells** | 0.272 | | <0.001 | |
|  |  | **NK CD56bright cells** | 0.153 | | 0.003 | |
|  | **Negative** | **TReg** | -0.164 | | 0.001 | |
|  |  | **pDC** | -0.344 | | <0.001 | |
|  |  | **Neutrophils** | -0.184 | | <0.001 | |
|  |  | **Mast cells** | -0.131 | | 0.011 | |
|  |  | **DC** | -0.226 | | <0.001 | |
|  |  | **Cytotoxic cells** | -0.278 | | <0.001 | |
|  |  | **CD8 T cells** | -0.166 | | 0.001 | |
| RBM15B | **Positive** | **Th2 cells** | 0.350 | | <0.001 | |
|  |  | **TFH** | 0.165 | | 0.001 | |
|  |  | **Tcm** | 0.104 | | 0.045 | |
|  |  | **T helper cells** | 0.269 | | <0.001 | |
|  |  | **NK CD56bright cells** | 0.150 | | 0.004 | |
|  | **Negative** | **TReg** | -0.226 | | <0.001 | |
|  |  | **Tgd** | -0.189 | | <0.001 | |
|  |  | **pDC** | -0.312 | | <0.001 | |
|  |  | **Neutrophils** | -0.208 | | <0.001 | |
|  |  | **Mast cells** | -0.114 | | 0.028 | |
|  |  | **DC** | -0.329 | | <0.001 | |
|  |  | **Cytotoxic cells** | -0.328 | | <0.001 | |
|  |  | **CD8 T cells** | -0.116 | | 0.025 | |
| RBM15 | **Positive** | **Th2 cells** | 0.339 | | <0.001 | |
|  |  | **Th1 cells** | 0.135 | | 0.009 | |
|  |  | **TFH** | 0.123 | | 0.018 | |
|  |  | **Tcm** | 0.186 | | <0.001 | |
|  |  | **T helper cells** | 0.294 | | <0.001 | |
|  |  | **NK CD56bright cells** | 0.105 | | 0.042 | |
|  |  | **aDC** | 0.179 | | <0.001 | |
|  | **Negative** | **TReg** | -0.137 | | 0.008 | |
|  |  | **pDC** | -0.243 | | <0.001 | |
|  |  | **DC** | -0.216 | | <0.001 | |
|  |  | **Cytotoxic cells** | -0.165 | | 0.001 | |
| METTL3 | **Positive** | **Th2 cells** | 0.268 | | <0.001 | |
|  |  | **Tcm** | 0.231 | | <0.001 | |
|  |  | **T helper cells** | 0.404 | | <0.001 | |
|  |  | **NK CD56bright cells** | 0.136 | | 0.009 | |
|  |  | **Eosinophils** | 0.132 | | 0.011 | |
|  | **Negative** | **TReg** | -0.159 | | 0.002 | |
|  |  | **Tgd** | -0.121 | | 0.019 | |
|  |  | **T cells** | -0.114 | | 0.028 | |
|  |  | **pDC** | -0.365 | | <0.001 | |
|  |  | **Neutrophils** | -0.182 | | <0.001 | |
|  |  | **Mast cells** | -0.109 | | 0.034 | |
|  |  | **DC** | -0.295 | | <0.001 | |
|  |  | **Cytotoxic cells** | -0.351 | | <0.001 | |
| METTL14 | **Positive** | **Th17 cells** | 0.191 | | <0.001 | |
|  |  | **Tcm** | 0.401 | | <0.001 | |
|  |  | **T helper cells** | 0.387 | | <0.001 | |
|  |  | **Eosinophils** | 0.198 | | <0.001 | |
|  | **Negative** | **pDC** | -0.326 | | <0.001 | |
|  |  | **NK CD56bright cells** | -0.183 | | <0.001 | |
|  |  | **Macrophages** | -0.119 | | 0.021 | |
|  |  | **DC** | -0.173 | | <0.001 | |
|  |  | **Cytotoxic cells** | -0.185 | | <0.001 | |
| CBLL1 | **Positive** | **Th2 cells** | 0.223 | | <0.001 | |
|  |  | **Tcm** | 0.276 | | <0.001 | |
|  |  | **T helper cells** | 0.284 | | <0.001 | |
|  | **Negative** | **Tgd** | -0.112 | | 0.030 | |
|  |  | **T cells** | -0.122 | | 0.018 | |
|  |  | **pDC** | -0.297 | | <0.001 | |
|  |  | **Neutrophils** | -0.116 | | 0.025 | |
|  |  | **DC** | -0.274 | | <0.001 | |
|  |  | **Cytotoxic cells** | -0.326 | | <0.001 | |
|  |  | **CD8 T cells** | -0.108 | | 0.036 | |
